# Aggressive systemic mastocytosis with multiple organ involvement: a case report

**DOI:** 10.1093/omcr/omad095

**Published:** 2023-11-28

**Authors:** Effat Iranijam, Maryam Salimi, Mohammad Negaresh, Nima Javanshir

**Affiliations:** Department of Internal Medicine (Hematology Division), School of Medicine, Ardabil University of Medical Sciences, Ardabil, Iran; Department of Internal Medicine, School of Medicine, Ardabil University of Medical Sciences, Ardabil, Iran; Department of Internal Medicine, School of Medicine, Ardabil University of Medical Sciences, Ardabil, Iran; Faculty of Medicine, School of Medicine, Ardabil University of Medical Sciences, Ardabil, Iran

## Abstract

Systemic mastocytosis is a rare malignancy whose main diagnostic finding is the abnormal proliferation of clonal mast cells. In this report, a 63-year-old woman is presented who was referred to the emergency department with lower back pain. Due to the hypereosinophilia in blood tests, a bone marrow biopsy was performed, and except for the presence of a large number of mastocytes, no other pathologic findings were seen. Furthermore, the immunohistochemistry test showed positive CD117 and CD25 markers, and the patient’s platelet-derived growth factor receptor alpha test was positive. Hence, the patient was diagnosed with aggressive systemic mastocytosis. Treatment was initiated with the Cladribine regimen, but unfortunately, in the third course, the patient experienced bradycardia and loss of consciousness and expired. Systemic mastocytosis can manifest itself with non-cutaneous symptoms. Non-cutaneous symptoms do not rule out systemic mastocytosis as a differential diagnosis in patients with hypereosinophilia.

## INTRODUCTION

Systemic mastocytosis (SM) is a rare and unique neoplasm characterized by the abnormal proliferation of clonal mast cells. The affected patients mainly refer to medical centers complaining of skin lesions, anaphylaxis or both simultaneously. Cutaneous lesions are this disease’s most common symptoms, mainly appearing in the first six months of life [1]. The clinical manifestations of the disease are associated with the acute release of various mast cell mediators such as heparin, histamine, eosinophil chemotactic factor, slow reaction substance and 5-hydroxytryptamine (5-HT). The related symptoms are pruritus, episodic flushing, syncope, gastrointestinal symptoms, musculoskeletal pain and neuropsychiatric disorders [2]. Based on the World Health Organization (WHO) criteria for SM, the diagnosis is made when at least one major and one minor or three minor criteria are fulfilled. The criteria are as follows: major criteria are Multifocal dense infiltrates of mast cells (≥15 mast cells in aggregates) in bone marrow biopsies and/or in sections of another extracutaneous organ(s). and minor criteria consist of (i) ≥25% of all mast cells are atypical cells (type I or type II) on bone marrow smears or are spindle-shaped in mast cell infiltrates detected in sections of bone marrow or other extracutaneous organs; (b) KIT-activating KIT point mutation(s) at codon 816 or in other critical regions of KIT in bone marrow or another extracutaneous organ; (iii) Mast cells in bone marrow, blood or another extracutaneous organ express one or more of CD2 and/or CD25 and/or CD30; and (iv) baseline serum tryptase concentration > 20 ng/mL (in the case of an unrelated myeloid neoplasm, an elevated tryptase does not count as an SM criterion. In the case of a known HαT, the tryptase level should be adjusted. The aggressive SM (ASM) is diagnosed when SM criteria are evident, along with organ damage caused by SM and lack of mast cell leukemia or associated hematologic neoplasm [3]. ASM is a rare disease, with a global prevalence estimated to be 1 in 250 000 to 400 000 [4]. The low prevalence of mastocytosis, its unusual characteristics and its complications often hinder its diagnosis. That is why the actual prevalence of the disease might be even higher [5]. The treatment of mastocytosis involves controlling symptoms and monitoring the patients’ status continuously. Systemic symptoms might have a gastrointestinal, pulmonary, skeletal, cutaneous or cognitive origin [6].

### Case report

The patient presented in this study was a 63-year-old female referred to the emergency department of Emam Khomeini Hospital of Ardabil, Iran, in December 2021 with the chief complaint of lower back pain. The lower back pain began three months earlier, spreading to the right hip and anterior pelvis. The pain had a mechanical nature and was exacerbated when performing strenuous activities. Other symptoms she mentioned included reduced appetite, weight loss of about 5 kg during the last three months, constipation, dyspepsia without dysphagia and night sweats. She had a history of COVID-19 infection 9 months before and diabetes mellitus for 10 years. She mentioned no history of trauma. She took a metoprolol tablet of 25 mg daily, a metformin tablet of 500 mg twice daily and a pantoprazole tablet of 40 mg twice daily. In the familial history, her mother died of gastric cancer. Her vital signs were as follows: blood pressure = 120/80, respiratory rate = 16, body temperature = 37, pulse rate = 78. The patient was pale. There were no signs of skin lesions or atopic dermatitis. Physical, cardiac, respiratory and skin examinations were normal. However, the abdominal examination found a 2–3 cm length splenomegaly under the ribs. Moreover, the examination of the spine revealed the existence of tenderness in the L3 and L4 lumbar vertebrae.

Considering the lower back pain, the high age of the patient, and the presence of point tenderness, the patient was tested for malignancies, especially multiple myeloma, and infection on the first day of admission.

The medical tests that were performed on the patient were as follows:

WBC = 18 700 cu/mm (PMN = 6826/LYMPH = 1926/MONO = 224/EOS = 9724), HB = 13.2 g/dl [7–11], PLT = 171 000/ml (150–450 10^6^/ml), ESR = 82 mm/hr (<36.5), ALKP = 695 IU/L (64–306), Ca = 8.8 mg/dl (8.2–10.7), Wright = neg, Coombs wright = neg, TSH = 1.1 mIU/L (0.5–5.0), PTH = 33.5 pg/mL (10–55), PCR for COVID19 = Negative.

An endoscopy was performed regarding gastrointestinal symptoms such as reduced appetite and weight loss, as well as the history of malignancies in the patient’s mother. The results were indicative of antral gastritis. Pathological investigations revealed that it was eosinophilic gastritis. With the diagnosis of hypereosinophilia and eosinophilic gastritis, her treatment began with prednisolone. Since the symptoms continued, an abdominopelvic ultrasound was performed. The results indicated that besides a splenomegaly with a length of 171 mm and a homogeneous parenchymal echo, her sonography was normal and no sign of lymphadenopathy was seen.

Based on the patient’s symptoms and initial observations, she was admitted to the hospital for a bone marrow biopsy (BMB) and additional tests to screen for possible malignancies. The medical team also conducted mammography, bone scintigraphy and tumor marker tests. Based on the results obtained from mammography, breast parenchymal density was uniform, and tiny nodules existed in the lateral part of the left breast (BIRADS = 1). The serum protein electrophoresis (SPEP) showed a polyclonal antibodies pattern. The results of densitometry tests on cervical and thoracic vertebrae were all normal. However, in lumbar vertebrae and femurs, osteoporosis was detected.

**Figure 1:**
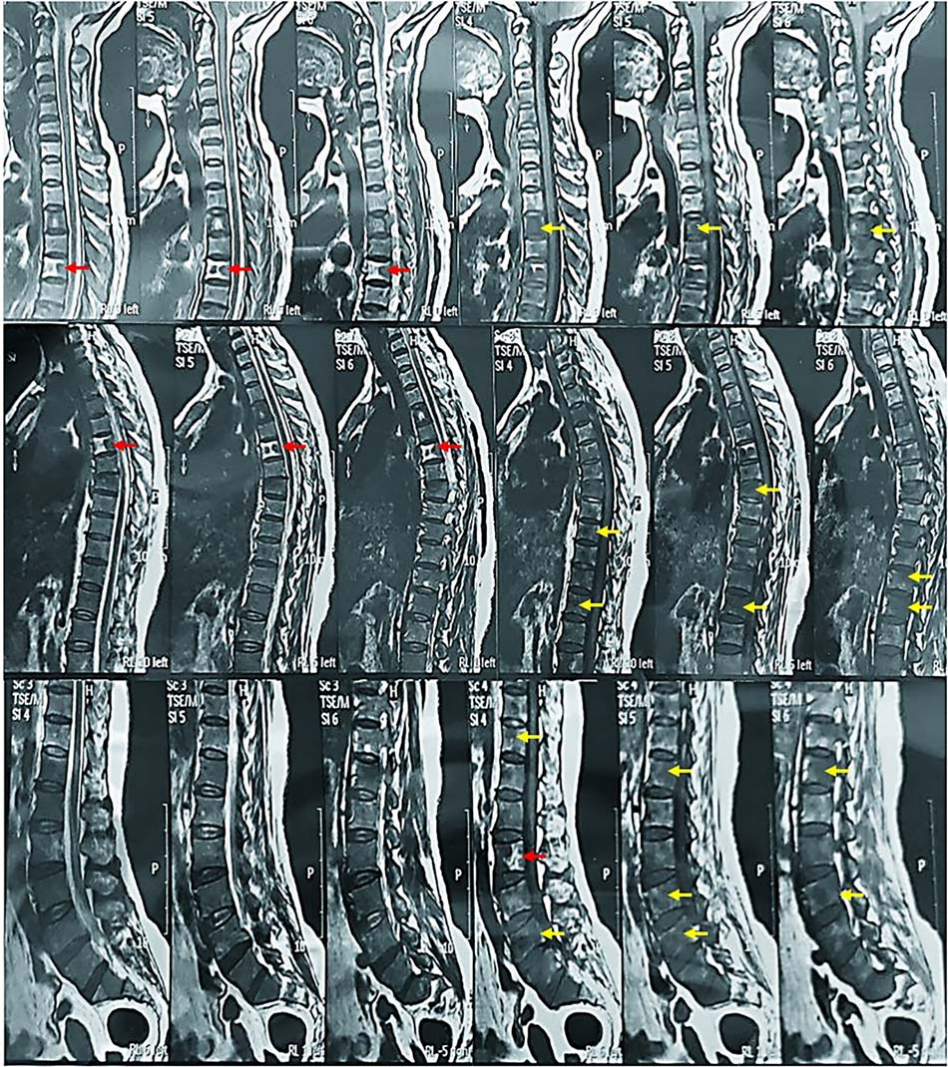
The patient’s MRI showed diffuse lytic (yellow arrow) and sclerotic (red arrow) lesions in the patient’s vertebras.

Her bone mineral densitometry was normal on cervical and thoracic vertebrae. However, in lumbar vertebrae and femurs, osteoporosis was detected. A whole-body bone scan was performed on the patient regarding the probability of bone metastases. The results indicated an increased radioactive uptake in ribs, vertebrae, bilateral pubis and the diaphysis of both femurs, favoring blood dyscrasia. Also, MRI revealed numerous lytic and sclerotic lesions in the cervical, thoracic and lumbar vertebras (Fig. 1). The patient’s tumor markers were as follows: CEA = 1.27 mg/L (up to 5.0), CA19–9(ECL) = 13.44 U/ml (up to 31.3), CA15–3(ECL) = 18.8 U/ml (up to 39), CA125(CL) = 12.5 U/ml (0–35), AFP = 1.05 U/ml (up to 35).

**Figure 2:**
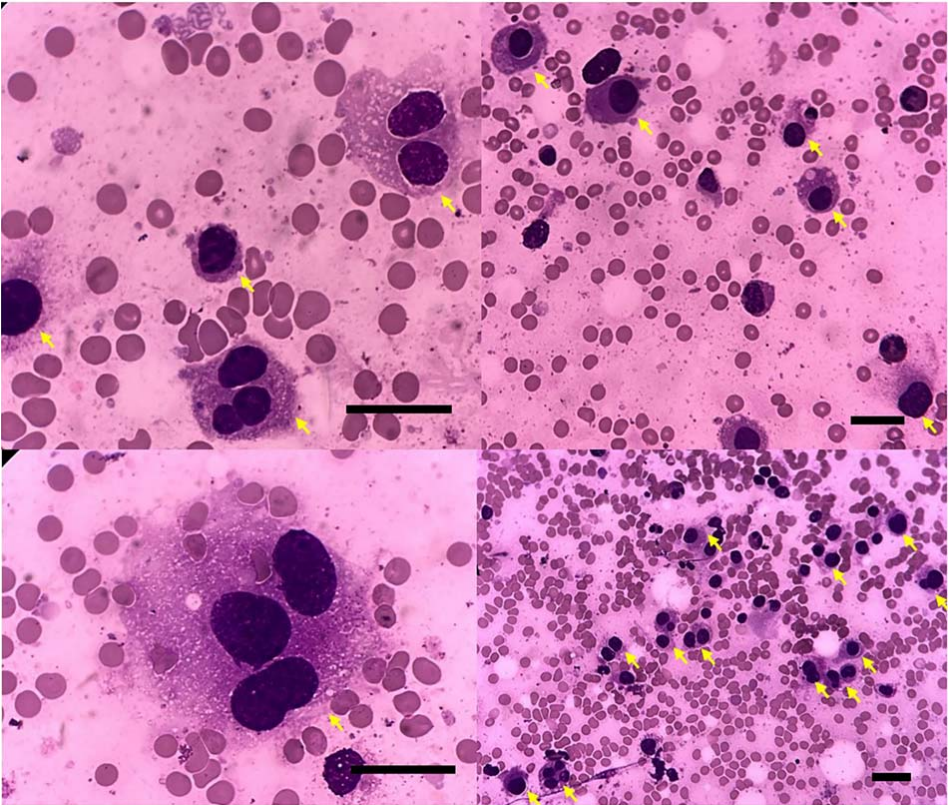
Patient’s bone marrow biopsy. Giant numerous round and ovoid mastocytes with dense chromatin and a large amount of dispersed coarse metachromatic granules in the cytoplasm (yellow arrow) consistent with systemic mastocytosis (bar = 50 um).

To check for hypereosinophilia, BMA and BMB were performed on the patient. The pathologist’s analysis of the slides revealed no other findings except that 17% of all nucleated cells were atypical mast cells (Fig. 2). Moreover, based on the results of the IHC test, CD117 and CD25 were reported to be positive. Molecular monitoring of PDGFRA was also requested, which was negative. The KIT mutation test was negative. Hence, the diagnosis of aggressive systemic mastocytosis was confirmed.

Further on, she developed bicytopenia (WBC = 5900 cu/mm (PMN = 1298/LYMPH = 590/MONO = 177/EOS = 3835), HB = 8.5 g/dl [7–11], PLT = 53 000/ml (150–450 10^6^/ml)) considering the night sweat, weight loss, splenomegaly, eosinophilia, bicytopenia, high ESR, osteoporosis of the vertebrae, eosinophilic gastritis, and the results of BMA/BMB and IHC tests, the final diagnosis of advanced systemic mastocytosis was made.

5 mg of prednisolone twice a day and 70 mg of alendronate once a week were prescribed for treating eosinophilic gastritis and osteoporosis. Furthermore, 7 days of chemotherapy with a Cladribine regimen with a daily dose of 90 μg/kg for systemic mastocytosis was prescribed. After the first session of chemotherapy, she was discharged.

In her second session of chemotherapy, she had gradually become ill. She had abdominal ascites, which continued to recur despite draining. A manual drain was inserted into the patient’s abdominal cavity to evacuate the ascites. The fluid was removed from her abdominal cavity once every other day and replaced with vials of albumin infusion. After that, the patient received the second cladribine course and was discharged.

She showed transient aphasia, mood and cognitive disorder in her third session, and a brain MRI was ordered. Based on the ischemic findings, the patient’s symptoms were justifiable in neurology consultation. The patient still had ascites. The tests also revealed that she had pancytopenia and a high LDH level. On the fourth day of receiving the Cladribine regimen, she developed bradycardia and loss of consciousness, and despite treatment measures, she became asystole and expired. The disease onset to expiry interval was seven months.

## DISCUSSION

In ASM the organ damages (C-findings) are Cytopenia which can be one or more of ANC < 1 × 109/L, Hb < 10 g/dL, PLT < 100 × 109/L, or hepatic symptoms like ascites and elevated liver enzymes with or without hepatomegaly or cirrhotic liver with or without portal hypertension, palpable splenomegaly with hypersplenism with or without weight loss and hypalbuminemia, gastrointestinal symptoms like malabsorption with hypoalbuminemia with or without weight loss, and skeletal symptoms of large osteolytic lesions (≥2 cm) with pathologic fracture with or without bone pain [3]. In this report, we presented a 63-year-old female referred to the hospital with systemic symptoms (weakness, night sweats and weight loss), gastrointestinal symptoms (nausea, vomiting and constipation) and lower back pain in the last 3 months without skin involvement. During hospitalisation, the patient developed bicytopenia, neurological symptoms (aphasia, mood disorder and cognitive disorder), and abdominal ascites.

The vertebrae’s involvement can be lytic, sclerotic or mixed in systemic mastocytosis. This specific case was identified to have a mixed pattern [12]. Abdominal pain is the most common symptom among patients suffering from SM, which occurs in 50%, nausea and vomiting in 30% and gastrointestinal bleeding in 10% of the affected cases. In addition, peptic ulcer has been reported in 25% of the patients, which is associated with mucosal and non-mucosal inflammatory infiltrates of mast cells and histamine secretion, a recognised stimulant of gastric acid secretion. Urticarial gastric lesions and eosinophilic gastritis have also been observed in several cases. These lesions are associated with mucosal and non-mucosal inflammatory infiltrates of mast cells [13]. The endoscopic revealed the presence of eosinophilic gastritis, which provided clues that the condition might be an allergic reaction or mastocytosis.

Our patient presented with hypereosinophilia with an eosinophil count of more than 1.5 × 109/L [14]. CD117 is related to normal mast cells, whereas CD25 is related to non-normal ones [15]. Considering that CD25 is a reliable immunohistochemical and diagnostic marker of SM [16], and both CD25 and CD117 were positive in our patient, the diagnosis of SM was made.

Based on the classification provided by the WHO for SM and considering the presence of symptoms such as cytopenia, hypoalbuminemia, malabsorption, osteolysis with or without pathological fractures, elevated liver enzymes and ascites in the patient under investigation, the final diagnosis was ASM [7]. In a study by Wolfgang et al., they observed that among the patients with SM, the prognosis of those who had organomegaly, higher age, higher levels of tryptase, LDH, alkaline phosphatase, and white blood cell count as well as lower hemoglobin concentration and lower platelet count was poorer [8]. The mentioned poor prognosis factors were mostly observed in our patient, which led to her early mortality after diagnosis and treatment initiation.

The treatment of SM involves controlling symptoms and monitoring the patients constantly. Systemic symptoms might have a gastrointestinal, pulmonary, skeletal, cutaneous or cognitive origin. To treat skin symptoms, including erythema, flushing, itching and blisters, histamine-2 receptor antagonists (H2) can be used [6]. For the treatment of itching, hydroxyzine or doxepin can be helpful. Gastrointestinal symptoms, including indigestion, can be treated with H2 receptor antagonists. Using cromolyn can reduce gastrointestinal symptoms (particularly diarrhea, abdominal pain and nausea) and alleviate skeletal pain and headache [9]. NSAIDs can also help minimise skeletal pain and irritating skin symptoms [6]. Steroids can be used minimally and cautiously in cases of severe mastocytosis due to their harmful effects on bone density [10]. A study by Jendoubi et al. evaluated the effectiveness of omalizumab in treating adults with mastocytosis. The results were promising, as omalizumab showed positive outcomes in treating life-threatening anaphylaxis and symptoms related to mast cells and improving patients’ quality of life [11]. In general, omalizumab is used for treating patients with severe symptoms who do not respond to leukotriene receptor antagonists or cromolyn. Calcium and vitamin D should be administered to patients with low bone density. Furthermore, the psychological health of the patients with SM should be attended to and improved as they are at a higher risk of depression. For this purpose, selective serotonin reuptake inhibitors can be used [6].

The Food and Drug Administration of the United States of America confirmed using Midostaurin to treat ASM on April 28th, 2017. It is a type of tyrosine kinase inhibitor for which a response rate of 60% has been reported in advanced SM cases. Midostaurin is well-tolerated by patients. Its side effects include low-grade nausea, vomiting and diarrhea. However, although this drug controls the disease in the first place, full recovery is not achieved in all patients [6]. As this drug was unavailable in Iran, our patient received a treatment regimen with Cladribine, the second most effective medication for treating advanced SM after midostaurin [17]. The outcome, however, was not satisfactory.

Our patient was referred to the hospital with the chief complaint of lower back pain, which is rare among patients with SM. This disease mainly manifests itself with skin symptoms. Such a condition regarding this patient caused a delay in diagnosing the disease.

It’s important to be aware that systemic mastocytosis can show non-cutaneous symptoms in its more aggressive forms. Even if there are no apparent typical symptoms, it’s crucial to consider this disease as a differential diagnosis in cases involving hypereosinophilia.

## Data Availability

The data supporting this study’s findings are available from the corresponding author, Nima Javanshir, upon reasonable request.
